# Human Alveolar Echinococcosis, Czech Republic, 2007–2014

**DOI:** 10.3201/eid2112.150743

**Published:** 2015-12

**Authors:** Libuše Kolářová, Jana Matějů, Jiří Hrdý, Hana Kolářová, Lubomíra Hozáková, Vita Žampachová, Herbert Auer, František Stejskal

**Affiliations:** National Reference Laboratory for Tissue Helminthoses, General University Hospital, Prague, Czech Republic (L. Kolářová, J. Matějů);; Charles University First Faculty of Medicine, Prague (L. Kolářová, J. Matějů, J. Hrdý, František Stejskal, H. Kolářová);; University Hospital, Ostrava, Czech Republic (L. Hozáková);; Masaryk University Faculty of Medicine, Brno, Czech Republic (V. Žampachová);; St. Anne’s University Hospital, Brno (V. Žampachová);; Medical University Vienna, Austria (H. Auer)

**Keywords:** *Echinococcus multilocularis*, tapeworm, cestodes, larva, human alveolar echinococcosis, hydatid disease, hydatidosis, echinococcal, neglected tropical disease, zoonoses, Czech Republic

**To the Editor:** Human alveolar echinococcosis (AE) is a zoonotic parasitic disease caused by larvae of *Echinococcus multilocularis* tapeworms that manifests most often in the host’s liver, although it can infect the lungs, brain, and other organs. Clinical symptoms usually develop after a long incubation period (5–15 years), causing considerable diagnostic difficulties and delay in treatment. The disease is reported in all countries neighboring the Czech Republic: Slovakia, Poland, Austria, and Germany (*1*,*2*). To complete data from central Europe, we present results on the occurrence of AE in the Czech Republic collected by the National Reference Laboratory for Tissue Helminthoses during 2007–2014.

In the Czech Republic, the occurrence of *E. multilocularis* in definitive (red fox, dog, cat, raccoon dog) and intermediate (bank vole) hosts was reported (*3*). After the first reports on detection of the parasites in foxes during 1995 (*4*; Figure), physicians started to request laboratory examinations for AE in persons with liver lesions, suspicious clinical symptoms, or both. During 1998–2014, examinations of 1,892 patients revealed 20 AE cases (12 women, 8 men); the first 2 cases were diagnosed during 2007 (*5*,*6*). In all cases, the diagnosis was based on AE characteristic imaging by using ultrasonography, computed tomography, magnetic resonance imaging, or a combination of these methods; in 19 (95%) cases, the results were confirmed by *E. multilocularis*–specific serology. In-house *E. multilocularis* crude–antigen was used for ELISA and Western blot testing and for ELISA IgG for detection of *E. multilocularis*. Em2–Em18 antibodies (Bordier Affinity Products SA, Crissier, Switzerland) were used for some laboratory examinations. Since 2009, in-house Western blot has been done by using a commercial set (LDBIO, Lyon, France).

**Figure:**
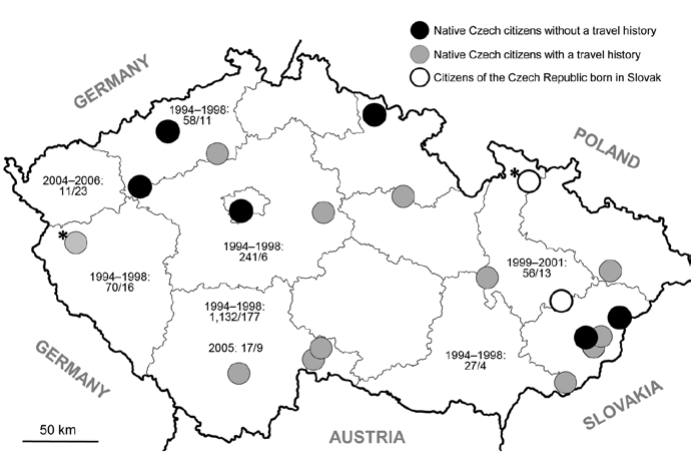
Distribution of human alveolar echinococcosis (AE) in the Czech Republic during 2007–2014, according to the site of residence of 20 case-patients, including their travel history. Asterisks (*) indicate AE cases already published (*6*,*7*). Six patients reported no travel outside the country; 2 patients were born in Slovakia and lived in the Czech Republic for 5 and 14 years before the time of initial AE diagnosis; the remaining patients traveled from the Czech Republic to various countries, including those to which AE is endemic. Nonperiodic examinations of red foxes (*4*,*9*) revealed the presence of *Echinococcus multilocularis* in the country. Date ranges indicate the period of examination; numbers separated by virgules indicate the number of foxes examined and those that tested positive, respectively.

In 18 cases, AE was also confirmed by characteristic histopathologic findings, species–specific molecular analysis of tissue biopsies, or both. PCR assay according to Schneider et al. (*7*) has been used in the National Reference Laboratory since 2011. In 17 (85%) patients, the liver was the only affected organ, and the infection was classified according to Brunetti et al. (*8*) as PN0M0; in 3 patients, liver and brain (PN0M1), retroperitoneum (PN1M0), or kidneys (PN1M0) were also affected.

Analysis of gender and age at the time of initial AE diagnosis showed that the youngest and the oldest patients were 21 and 82 years old, respectively. To examine differences in non–Gaussian distributed variables between male and female patients, we used the 2-way Mann–Whitney nonparametric test (GraphPad, San Diego, CA, USA) to analyze age data. The mean and median age of patients were lower among women (mean 45, median 36.5 years) than in men (mean 53, median 60 years), but these differences were not statistically significant (p˃0.05).

According to the site of residence, the patients originated from different parts of the Czech Republic (Figure). The disease was diagnosed in 18 native Czech citizens and in 2 citizens from Slovakia.

Physicians interviewed 17 of 20 patients in whom AE was diagnosed and completed questionnaires with patient data including clinical signs and symptoms, laboratory findings, and medical history (e.g., job, hobbies, travels abroad, ownership of animals) at the time of the first medical visit before diagnosis. Ten patients reported a prickling sensation and abdominal discomfort or pain. Three patients palpated a solid mass in the right hypochondrium before physical examination. Another 3 patients reported fever, fatigue, or malaise; 1 patient reported weight loss. The first clinical examinations by physicians revealed hepatomegaly in 16 patients; in addition, 3 of these patients had anemia and 1 had jaundice.

In the medical history, dog or cat ownership, gardening, farming, or hunting were recorded in some cases, which is similar to what was reported by Kern et al. (*10*). Of 15 persons interviewed who were native to the Czech Republic, 6 reported no travel outside the country (Figure). Because of the occurrence of *E. multilocularis* in animals (Figure), we assume that AE may have a characteristic of autochthonous infection in the Czech Republic. The 2 patients from Slovakia lived in the Czech Republic for 5 (*5*) and 14 years, respectively, before the diagnosis of AE. Considering the long incubation period of the disease, these patients were likely infected in Slovakia, where occurrence of AE is also reported (*1*).

In summary, we report 20 cases of human AE in the Czech Republic during 1998–2014. However, because asymptomatic patients with only mild liver involvement are unlikely to seek clinical investigation, the actual number of patients in the Czech Republic who have AE is expected to be even higher than that reported here.
